# Scientific agency in preschool STEM: a play-based developmental model

**DOI:** 10.3389/fpsyg.2026.1868005

**Published:** 2026-06-22

**Authors:** Ahmet Sami Konca, Hüseyin Ateş

**Affiliations:** 1 Department of Learning and Educational Leadership, College of Education, United Arab Emirates University, Al Ain, United Arab Emirates; 2 Department of Science Education, Kırşehir Ahi Evran Üniversitesi, Kırşehir, Türkiye

**Keywords:** classroom emotional climate, early childhood education, embodied cognition, epistemic curiosity, play-based inquiry, preschool STEM, scientific agency, teacher mediation

## Abstract

Early childhood STEM is often justified through future achievement or described through activities and materials, yet these accounts do not fully explain how playful exploration becomes child-authored inquiry. This article develops a conceptual account of scientific agency in preschool STEM: a young child’s situated capacity to influence how a phenomenon is questioned, investigated, interpreted, and revisited with others. The argument addresses a gap in activity-centered and outcome-oriented accounts of early STEM, which often document exposure, engagement, or intervention effects without specifying when hands-on experience becomes epistemic participation. The proposed Play-Based STEM Agency Model is a theoretically derived framework rather than the result of a single empirical intervention. It synthesizes early childhood pedagogy, constructivist and sociocultural learning theory, curiosity research, embodied cognition, and science education work on inquiry practices. The model proposes a recursive pathway in which play-based STEM inquiry creates accessible uncertainty; uncertainty invites epistemic curiosity; embodied investigation makes ideas testable through action and material feedback; and meaning-making connects experience with comparison, representation, explanation, and revision. Scientific agency emerges when children’s contributions shape the direction of inquiry and are recognized by teachers and peers. By specifying mechanisms, moderators, and boundary conditions, the model generates testable hypotheses and offers process-sensitive indicators for research, teacher education, and assessment in preschool STEM.

## Introduction

Interest in early childhood STEM education has expanded across policy, curriculum, and research contexts. Recent reviews and bibliometric analyses indicate that this expansion has been accompanied by uneven attention to early years, assessment, cultural context, and non-Western settings (Bui et al., 2024; Revák et al., 2024; Roy et al., 2025; Wan et al., 2021). Science, technology, engineering, and mathematics are increasingly introduced before formal schooling becomes strongly disciplinary, often because early experiences are expected to support later academic learning, scientific literacy, and participation in a technologically complex society (Bybee, 2013; McClure et al., 2017; National Research Council, 2012). These future-oriented arguments have helped make STEM more visible in early childhood education. Yet they do not fully explain why STEM matters for young children in the present, nor do they specify what makes a preschool STEM experience educationally meaningful.

This issue is especially important because preschool education is not a simplified version of later schooling. In the preschool years, children learn through play, language, embodied action, social interaction, symbolic representation, and emerging self-regulation (Copple and Bredekamp, 2009; National Association for the Education of Young Children, 2020; Vygotsky, 1978). Young children rarely approach the world through formal disciplinary categories. They notice that wet sand holds a tower differently from dry sand, that a bridge bends when more objects are placed on it, that a shadow changes when a light source moves, or that one ball rolls farther than another. Such episodes can become foundations for scientific and mathematical reasoning when children are supported to observe, compare, predict, represent, explain, and revise their ideas (Eshach and Fried, 2005; Gelman et al., 2009; Gopnik, 2012; Jirout and Klahr, 2012).

Developmental and early science research has shown that young children can search for regularities, explore causal relations, generate explanations, and learn from evidence when environments make these processes accessible (Legare, 2014; Schulz and Bonawitz, 2007). Early childhood science education similarly emphasizes that preschool classrooms can support observing, predicting, comparing, representing, and discussing phenomena when activities are developmentally appropriate and socially supported (French, 2004; Konca et al., 2024; Worth, 2010). The central problem, therefore, is not whether young children are capable of STEM-related reasoning. The more precise problem is how playful and hands-on experiences become epistemically meaningful.

Hands-on activity is not automatically epistemically productive. A classroom may include ramps, magnets, coding toys, blocks, water tables, planting activities, measuring tools, or design challenges while offering limited opportunity for children to pose problems, test alternatives, interpret evidence, or change the direction of investigation. Conversely, a modest activity can become intellectually rich when children compare outcomes, use representations, discuss possible explanations, and influence what the group investigates next. Existing work has generated important knowledge about early STEM activities, teacher knowledge, classroom materials, and intervention outcomes. Less attention has been given to the psychological mechanism through which these experiences become child-authored inquiry.

Several established approaches address parts of this problem but leave the full mechanism underspecified. Guided play research shows that adult support and child autonomy can coexist, but it does not always specify when guidance supports evidence-oriented STEM inquiry rather than task completion or enjoyable exploration (Hirsh-Pasek et al., 2009; Weisberg et al., 2013; Zosh et al., 2017). Inquiry-based science education identifies practices such as questioning, predicting, testing, representing, and explaining, yet these practices can become procedural checklists when applied rigidly to preschool settings (National Research Council, 2007, 2012). Integrated STEM research emphasizes cross-domain connections, design challenges, and teacher professional learning, but the child’s epistemic position within inquiry often remains secondary (Campbell et al., 2018; MacDonald et al., 2020; Wan et al., 2021). Science identity frameworks illuminate recognition, competence, performance, and belonging, but they are oriented toward longer-term identity development rather than the episode-level participation through which preschool children first experience themselves as contributors to inquiry (Carlone and Johnson, 2007).

This article proposes scientific agency as a developmentally appropriate construct for addressing that explanatory gap. Scientific agency refers to preschool children’s situated capacity to participate as authors of inquiry: to influence what is noticed, what is tested, how evidence is interpreted, and how a shared investigation continues. The construct is narrower than general engagement, because engaged children may still follow an adult script. It is also distinct from self-efficacy, because feeling capable does not necessarily mean that one’s ideas shape classroom sense-making. It is more immediate than science identity, because preschool children can contribute meaningfully to inquiry before forming longer-term narratives of belonging in science.

To explain how such agency develops, the article proposes the Play-Based STEM Agency Model. The model is not derived from a single empirical intervention and should not be read as already empirically validated as a complete system. It is a theoretically derived and testable conceptual synthesis that brings together early childhood developmental pedagogy, constructivist and sociocultural accounts of learning, curiosity research, embodied cognition, and science education work on inquiry practices (Dewey, 1938; Piaget, 1952; Vygotsky, 1978; Barsalou, 2008; Jirout and Klahr, 2012; National Research Council, 2007, 2012). Its purpose is to clarify how established constructs can be organized around a specific preschool STEM outcome: children’s recognized influence within inquiry.

The model proposes a recursive relation among five components. Play-based STEM inquiry provides a developmentally appropriate context in which adults design materials, time, and discourse while preserving children’s authorship. Epistemic curiosity gives children a reason to pursue uncertainty, especially when events are surprising, incomplete, or discrepant. Embodied investigation allows children to test ideas through movement, manipulation, perception, and material feedback. STEM meaning-making connects action to comparison, representation, explanation, and revision. Scientific agency emerges when children’s contributions become consequential for the direction and interpretation of inquiry.

This process is situated within classroom and cultural conditions. Teacher beliefs, teacher self-efficacy, and classroom emotional climate shape whether uncertainty is preserved, whether children’s partial ideas are treated as worth pursuing, and whether failure becomes a resource for revision rather than a reason to stop. Broader boundary conditions also matter. Culture influences norms about play, questioning, adult authority, error, and peer talk. Developmental appropriateness determines whether selected STEM concepts and tools are accessible to preschool children. Material and institutional conditions shape whether teachers have the time, space, staffing, and resources needed to sustain inquiry.

The remainder of the article proceeds in six steps. First, it explains why preschool STEM requires a psychological theory that protects developmental appropriateness while clarifying what makes activity epistemically meaningful. Second, it presents the theoretical grounding and development of the Play-Based STEM Agency Model. Third, it defines scientific agency and differentiates it from interest, engagement, self-efficacy, inquiry participation, epistemic participation, general agency, and science identity. Fourth, it presents the model’s core components, contextual moderators, and boundary conditions. Fifth, it distinguishes the model from adjacent approaches and formulates testable hypotheses. Finally, it outlines implications for research, teacher education, assessment, and future empirical work.

## Why preschool STEM needs a psychological theory

The case for preschool STEM is often made through future-oriented claims: early STEM is expected to prepare children for school, later coursework, scientific literacy, and participation in a technologically complex society (Bybee, 2013; McClure et al., 2017; National Research Council, 2012; Wan et al., 2021). These claims are important, but they are incomplete for early childhood education. Preschool STEM also requires an account of what makes STEM meaningful for young children in the present. Throughout this article, *preschool* refers primarily to children between approximately 3 and 6 years of age, while recognizing that early childhood is often defined more broadly as birth through age eight. This narrower focus matters because preschool learning is organized through play, embodied action, language, social participation, representation, and emerging self-regulation rather than through formal disciplinary instruction (Copple and Bredekamp, 2009; National Association for the Education of Young Children, 2020; Vygotsky, 1978).

A psychological theory is therefore needed because the central issue is not only whether STEM materials or activities are available. The more precise issue is how children encounter phenomena, experience uncertainty, act on materials, coordinate observations with language and representation, and come to see their own ideas as consequential for inquiry. Educational psychology provides constructs for explaining this process: developmental appropriateness, curiosity, motivation, embodiment, social mediation, classroom climate, teacher beliefs, and children’s emerging agency. In the Play-Based STEM Agency Model, these constructs are not added as separate topics. They clarify how playful activity can become child-authored, evidence-oriented, and developmentally meaningful inquiry.

## Developmental appropriateness

A first reason for a psychological theory is developmental appropriateness. Preschool STEM should not be treated as a simplified version of elementary science, mathematics, engineering, or technology instruction. Young children usually encounter STEM through everyday phenomena rather than through formal disciplinary categories. They notice that wet sand holds a tower differently from dry sand, that a bridge bends when more objects are placed in the middle, that a shadow changes when a light source moves, that seeds grow unevenly, or that one object rolls farther than another. These episodes become STEM learning when children are supported to compare, predict, test, represent, explain, and revise their ideas (Eshach and Fried, 2005; French, 2004; Gelman et al., 2009; Gopnik, 2012; Jirout and Klahr, 2012; Worth, 2010).

Developmental appropriateness is therefore not only a matter of choosing simpler content. It also concerns the form of inquiry made available to children. A topic is developmentally appropriate when its relevant relations can be perceived, manipulated, discussed, represented, and revisited by preschool learners. Balance, motion, growth, pattern, quantity, stability, cause, evidence, and design constraint can all be accessible when they are embedded in play and supported through concrete materials and responsive talk. By contrast, an activity may be labeled STEM but remain developmentally weak if children merely follow adult directions, complete a product, or reproduce terminology without having opportunities to investigate relations among objects, actions, and outcomes.

Piagetian and Vygotskian perspectives help explain why this distinction matters. From a Piagetian perspective, children construct knowledge through active manipulation and through encounters with outcomes that do not fit existing expectations. When a tower collapses despite appearing stable, or when a ball rolls more slowly than expected, the discrepancy can create disequilibrium and invite accommodation of prior understandings (Piaget, 1952). However, discrepancy alone does not guarantee learning. Vygotsky’s account of mediation explains how adult and peer interaction can make such moments socially and linguistically productive. Teacher prompts, peer comparison, shared tools, and classroom language can help children transform a surprising outcome into something that can be named, compared, tested, and discussed (Vygotsky, 1978; Wood et al., 1976). Piagetian disequilibrium helps explain why discrepant outcomes can invite cognitive reorganization, whereas Vygotskian mediation explains how adult and peer interaction make such disequilibrium socially and linguistically productive.

In the Play-Based STEM Agency Model, developmental appropriateness functions as a boundary condition and design principle. Play-based STEM inquiry is not developmentally appropriate simply because it is playful or hands-on. It becomes appropriate when the environment allows children to act on phenomena, notice consequential differences, encounter manageable uncertainty, and participate in meaning-making with others. This is why preschool STEM should be designed less as downward extension of later school science and more as guided participation in inquiry around phenomena children can perceive, manipulate, question, and interpret.

## Motivation and epistemic curiosity

A second reason for a psychological theory concerns motivation. Preschool children often show strong interest in novelty, movement, materials, and social interaction. Yet spontaneous interest does not automatically become sustained inquiry. A child may be attracted to a shiny object, repeat an enjoyable action, or move quickly from one material to another without comparing outcomes, seeking evidence, or constructing explanations. Educational psychology helps distinguish between activity, engagement, and inquiry-oriented motivation. In educational settings, motivation is shaped by how environments support autonomy, competence, relatedness, persistence, and meaningful participation (Deci and Ryan, 2000; Schunk and DiBenedetto, 2020).

This distinction is especially important for epistemic curiosity. Curiosity can refer broadly to interest in novelty, but the form most relevant to preschool STEM is curiosity directed toward explanation and understanding (Engel, 2011; Jirout and Klahr, 2012; Loewenstein, 1994). Three forms should be differentiated. *Novelty curiosity* refers to attraction to something new or perceptually interesting. *Situational interest* refers to temporary engagement generated by an activity, material, or social context. *Epistemic curiosity* refers to the desire to reduce an explanation gap, resolve uncertainty, or understand why something happened. In this model, epistemic curiosity refers not to general attraction to novelty but to children’s motivation to reduce an explanation gap: why a tower collapsed, why one object rolled farther, or why a plant bent toward light.

The word *epistemic* is important because it identifies the knowledge-seeking quality of curiosity. A child who simply enjoys rolling balls down a ramp is active and possibly engaged. A child who asks why one ball traveled farther, changes the surface, repeats the trial, and compares the results is showing curiosity oriented toward evidence and explanation. This curiosity supports scientific agency because it gives children a reason to initiate or continue inquiry. They ask for another trial, test an alternative, notice an anomaly, revise an explanation, or invite peers to compare what happened.

Uncertainty is therefore central, but it must be manageable and socially supported. A collapsing tower, leaking container, unexpected shadow, or unsprouted seed can invite investigation when children experience puzzlement as safe and productive. The same uncertainty can also lead to withdrawal if children feel evaluated, rushed, or corrected too quickly. Research on exploratory play and early scientific reasoning shows that children often explore more when evidence is uncertain, ambiguous, or confounded, particularly when the context allows them to investigate rather than simply receive an answer (Schulz and Bonawitz, 2007). For preschool STEM, the educational question is therefore not how to remove uncertainty, but how to preserve it in ways that invite comparison, testing, representation, and explanation.

This clarification also protects preschool STEM from product-oriented or worksheet-driven implementation. Documentation, drawing, counting, photographing, measuring, and charting can deepen inquiry when they help children revisit and interpret their ideas. They become less valuable when visible products replace inquiry processes or when children complete predetermined answers without investigating phenomena. In the Play-Based STEM Agency Model, epistemic curiosity functions as the motivational mechanism that links play-based inquiry to children’s active investigation. Play creates the context, but epistemic curiosity explains why children pursue uncertainty as something to understand.

## Teacher-mediated inquiry ecology

A third reason for a psychological theory is teacher mediation. Early STEM learning cannot be explained by child behavior or classroom materials alone. Teachers arrange materials, allocate time, decide when to ask or wait, interpret children’s questions, distribute opportunities to speak, and determine whether a failed attempt is treated as an error or as evidence. Research on early childhood STEM emphasizes the importance of teacher knowledge, pedagogical content knowledge, beliefs, confidence, and professional learning for shaping the quality of STEM experiences (Campbell et al., 2018; Chen et al., 2025; MacDonald et al., 2020; Wan et al., 2021). Teacher talk also matters because prompts, revoicing, elaboration, and questions can help children connect action with comparison, representation, and explanation (Studhalter et al., 2021).

The term *inquiry ecology* is used here descriptively to refer to the arrangement of materials, time, teacher discourse, peer interaction, classroom norms, and institutional expectations that make inquiry more or less possible. It is not introduced as a new grand theory. It is an analytic term for the conditions through which playful activity becomes—or fails to become—STEM inquiry. The same materials can support very different forms of learning depending on this ecology. Blocks, ramps, plants, water containers, measuring tools, or coding toys do not themselves determine whether children will engage in inquiry. The difference lies in how the environment is framed, how uncertainty is handled, how children’s ideas are taken up, and whether the classroom allows repeated attempts and shared interpretation.

Teacher guidance must therefore be carefully specified. Guidance does not mean controlling every step of play or leading children toward one predetermined answer. It means designing affordances that make relations visible while preserving children’s authorship. A prompt such as “What do you notice about how far these travel?” functions as guidance because it orients attention toward comparison without specifying which object to choose, which outcome to produce, or which explanation to accept. Children retain authorship by selecting materials, proposing trials, changing surfaces, repeating actions, and interpreting what happened. The teacher supports inquiry by keeping the phenomenon discussable, by helping children compare outcomes, and by treating their ideas as possible directions for further investigation.

This form of mediation is consistent with sociocultural accounts of learning. Vygotsky’s theory emphasizes that development is shaped through tools, language, interaction, and participation in culturally organized activity (Vygotsky, 1978). Scaffolding further clarifies how adults can support learners by structuring a task, focusing attention, maintaining motivation, and gradually transferring responsibility as children become more capable participants (Wood et al., 1976). In preschool STEM, such mediation is not a temporary addition to learning; it is part of the ecology through which children’s actions become interpretable as inquiry. A child’s gesture, trial, or explanation becomes educationally consequential when it is noticed, named, compared, taken up by peers, or used to decide what the group will test next.

In the Play-Based STEM Agency Model, teacher-mediated inquiry ecology explains why materials and activities alone are insufficient. Play-based inquiry generates opportunities for uncertainty, but teachers and peers help determine whether that uncertainty becomes epistemic curiosity, whether embodied action becomes interpretable, and whether meaning-making supports scientific agency. A classroom ecology that preserves uncertainty, normalizes revision, supports representation, and recognizes children’s contributions makes it more likely that children will experience themselves as capable participants in inquiry. For this reason, preschool STEM needs psychological theory not to make play more formal, but to explain how play, motivation, action, mediation, and recognition work together in the development of scientific agency.

## Theoretical grounding and model development

The Play-Based STEM Agency Model is proposed as a theoretically derived and testable conceptual model rather than as an empirically validated model. It is not the outcome of a single intervention study, dataset, or classroom experiment. Instead, it synthesizes five bodies of scholarship that are often discussed separately in early childhood and science education: developmentally appropriate early childhood pedagogy, constructivist and sociocultural theories of learning, research on epistemic curiosity, embodied cognition, and science education accounts of inquiry and evidence use. The purpose of the model is to organize these established traditions around a more specific developmental question: how playful preschool STEM experiences become child-authored inquiry.

The model begins from early childhood pedagogy because preschool STEM must be interpreted through the forms of learning most appropriate for young children. Preschool children do not usually encounter science, technology, engineering, and mathematics as formal disciplinary systems. They encounter phenomena through play, materials, bodily action, language, social interaction, and representation. Developmentally appropriate practice therefore provides the boundary for the model: STEM learning should involve relations that children can perceive, manipulate, discuss, represent, and revisit with others (Copple and Bredekamp, 2009; National Association for the Education of Young Children, 2020).

Constructivist and sociocultural theories explain why activity alone is insufficient. Piagetian constructivism highlights active manipulation, disequilibrium, cognitive conflict, assimilation, and accommodation. When children encounter an unexpected outcome, such as a tower collapsing or a ball rolling differently across two surfaces, the discrepancy can invite reorganization of prior understanding (Piaget, 1952). Vygotskian sociocultural theory explains how such moments become socially and linguistically productive. Adult mediation, peer interaction, classroom tools, and shared language help transform material action into something that can be named, compared, tested, and discussed (Vygotsky, 1978; Wood et al., 1976). In this sense, Piagetian disequilibrium explains why discrepant outcomes may invite cognitive reorganization, whereas Vygotskian mediation explains how adult and peer interaction make such disequilibrium available for shared inquiry.

Research on curiosity provides the motivational logic of the model. Preschool children may be attracted to novelty, but STEM inquiry requires more than perceptual interest. The form of curiosity most relevant here is epistemic curiosity: the desire to reduce uncertainty, resolve an explanation gap, or understand why an event occurred (Engel, 2011; Jirout and Klahr, 2012; Loewenstein, 1994). This literature explains why uncertainty is central to the model. A surprising or incomplete event can motivate children to ask questions, repeat trials, compare outcomes, and seek explanations when the classroom treats uncertainty as safe and investigable.

Embodied cognition provides the investigative logic. Preschool children often think through action before they can fully explain their reasoning verbally. They push, pour, stack, rotate, sort, count, gesture, and reposition materials to test possibilities. Theories of embodied and grounded cognition suggest that conceptual activity is situated in perception, movement, and material contexts (Barsalou, 2008; Wilson, 2002). For preschool STEM, this means that action on materials is not merely preparation for thought; it is part of how children form, externalize, and revise ideas.

Science education frameworks provide the domain-specific practices that distinguish STEM inquiry from generic play. Observing, predicting, testing, comparing, representing, explaining, using evidence, and revising ideas are central practices in science learning (National Research Council, 2007, 2012). However, in preschool classrooms these practices must be developmentally transformed. They should not become rigid procedural steps. Rather, they should appear as flexible forms of guided participation: noticing what changed, trying a different material, drawing what happened, comparing two outcomes, or explaining why a new design worked better.

The five components of the model were selected because each performs a distinct theoretical function. Play-based STEM inquiry functions as the pedagogical entry condition: it creates a child-authored but adult-supported problem space. Epistemic curiosity functions as the motivational mediator: it explains why children pursue uncertainty rather than abandon it. Embodied investigation functions as the investigative mechanism: it allows children to test ideas through movement, manipulation, and material feedback. STEM meaning-making functions as the interpretive mechanism: it connects action with comparison, representation, explanation, and revision. Scientific agency functions as the proximal developmental outcome and recursive driver: it emerges when children’s contributions influence the direction of inquiry and are recognized by teachers and peers.

The model operates across multiple levels. At the child level, it concerns curiosity, embodied action, explanation, persistence, and inquiry authorship. At the teacher level, it concerns beliefs, self-efficacy, discourse, scaffolding, and the recognition of children’s ideas. At the classroom level, it concerns emotional climate, peer participation, materials, time, and norms for uncertainty and revision. At the cultural and institutional level, it concerns broader expectations about play, adult authority, questioning, error, curriculum, assessment, resources, and participation. These levels are not separate layers added to the model after the fact. They define the ecology in which preschool STEM agency becomes more or less possible.

The novelty of the model therefore does not lie in claiming that play, curiosity, embodiment, inquiry, or teacher mediation are new constructs. Its contribution lies in assigning these constructs distinct functional roles within a developmental account of preschool STEM. The model asks not only whether children participate in STEM activities, but whether classroom conditions allow them to influence what is questioned, tested, interpreted, and revisited. In doing so, it shifts the analytic focus from STEM activity provision to children’s recognized authorship within inquiry.

## Core construct: scientific agency

Scientific agency refers to preschool children’s situated and socially recognized capacity to influence STEM inquiry by shaping what is noticed, questioned, tested, interpreted, and revisited. This definition is intentionally developmentally modest. It does not require young children to use formal scientific vocabulary, complete a full experimental sequence, or describe themselves as scientists. Instead, it focuses on whether children participate as authors of inquiry: noticing a problem, proposing a test, changing a material arrangement, comparing outcomes, offering an explanation, revising an idea, or influencing what the group investigates next.

The construct is grounded in educational psychology and science education, but it is not reducible to any single tradition. Social cognitive theory conceptualizes agency as intentional action, self-reflection, and influence on one’s environment (Bandura, 2001). In preschool STEM, these features appear in developmentally emergent forms when children choose a strategy, repeat a trial, explain an outcome, or alter a design after feedback. Research on agentic engagement similarly shows that learners can shape learning conditions by asking questions, expressing preferences, and contributing to the direction of activity (Reeve, 2013). For preschool children, such contributions may be verbal, gestural, material, or collaborative.

Sociocultural theory is especially important because scientific agency is not treated here as an individual trait located only inside the child. Vygotsky’s account of mediation suggests that children’s participation develops through language, tools, interaction, and culturally organized activity (Vygotsky, 1978). A child’s action becomes consequential when it is noticed, named, revoiced, compared, or taken up by others. Scientific agency is therefore relational. It depends not only on what a child does, but also on whether teachers and peers recognize the child’s contribution as relevant to inquiry.

Science education gives the construct its domain specificity. Scientific agency is not general assertiveness, choice-making, or classroom participation. It is agency within practices of inquiry: observing, predicting, testing, comparing, representing, explaining, using evidence, and revising ideas (National Research Council, 2007, 2012). A child who chooses a crayon color may be exercising general agency. A child who changes the base of a block tower because the earlier design collapsed is exercising scientific agency, provided that the action is connected to evidence, explanation, or further investigation.

Scientific agency is not simply interest in STEM materials, persistence during an activity, confidence in one’s ability, or participation in an inquiry sequence. It refers specifically to a child’s authored and socially recognized contribution to the direction and interpretation of inquiry. This negative definition is necessary because several adjacent constructs overlap with scientific agency without being equivalent to it.

Interest describes attraction, preference, or valuing. Children can be interested in magnets, ramps, water, plants, blocks, or digital tools without influencing the trajectory of inquiry. Behavioral engagement describes attention, participation, effort, or time on task (Fredricks et al., 2004). Children can be highly engaged while following an adult script. Self-efficacy concerns perceived capability (Bandura, 2001; Riggs and Enochs, 1990). A child may feel capable but still have little influence on what the classroom investigates or how an outcome is interpreted. Inquiry participation means taking part in practices such as predicting, testing, observing, or recording, but these practices may still be adult-directed. Scientific agency asks whether children help author those practices.

The construct is also related to, but distinct from, epistemic participation and science identity. Epistemic participation refers broadly to involvement in knowledge-building activity. Scientific agency is narrower: it concerns whether a child’s contribution becomes consequential for the direction, evidence use, or interpretation of STEM inquiry. Science identity frameworks emphasize recognition, competence, performance, belonging, and longer-term self-understanding in relation to science (Carlone and Johnson, 2007). Scientific agency is treated here as a proximal, episode-level form of epistemic authorship that may contribute to later science identity, but it does not require preschool children to have already formed stable self-descriptions as science learners.

Scientific agency can be analyzed through three interrelated dimensions. Cognitive agency concerns children’s role in generating, testing, coordinating, and revising ideas about phenomena. It appears when children ask why or how questions, make predictions, notice relevant contrasts, compare outcomes, or change a strategy after evidence. Motivational agency concerns children’s willingness to enter uncertainty, persist after difficulty, and treat their own effort as meaningful for understanding. It appears when children return to an unresolved problem, request another trial, try again after failure, or treat a surprising outcome as worth investigating. Social agency concerns recognition and uptake. It appears when a teacher revoices a child’s idea, peers build on a child’s suggestion, or the group’s next action is shaped by a child-generated proposal.

Scientific agency should not be romanticized. It does not imply that all child ideas are equally adequate, that adult guidance is unnecessary, or that children should be left to discover everything independently. Preschool STEM agency is supported through carefully designed environments, contingent teacher mediation, and opportunities for shared meaning-making. Adults can preserve child initiative while still guiding attention toward comparison, evidence, representation, and explanation. The key issue is not whether adults intervene, but whether their mediation expands or replaces children’s authorship.

Because the construct is situated and observable, it can be operationalized without relying only on verbal reports. Indicators include child-generated questions, child-initiated tests or modifications, evidence-based comparisons, causal explanations, revisions after discrepant outcomes, requests for further investigation, and collaborative meaning-making. Nonverbal and multimodal indicators are especially important in preschool. A child may show reasoning by pointing to a relevant contrast, moving a heavier block to the base of a tower, repeating a ramp trial with a different surface, or gesturing the path of a shadow before naming it. Such actions should be interpreted as evidence of scientific agency when they influence inquiry and are connected to sense-making.

Positioning scientific agency at the center of preschool STEM bridges developmental and educational concerns. Developmentally, it recognizes that young children reason through action, language, representation, and interaction. Educationally, it gives pedagogy and research a more precise aim: to create conditions in which children are not merely present in STEM activities, but are recognized as contributors whose ideas can shape inquiry.

## The play-based STEM agency model

Building on the theoretical grounding and construct definition above, the Play-Based STEM Agency Model explains how preschool STEM experiences can support scientific agency through a recursive set of developmental, motivational, embodied, interpretive, and social processes. The model should not be read as a rigid sequence of instructional steps. Preschool inquiry often begins with action before language, with material exploration before an explicit question, or with a teacher’s revoicing of an idea after a child has already tested something. The model therefore identifies a patterned relation among components rather than a fixed instructional order.

**Figure 1:**
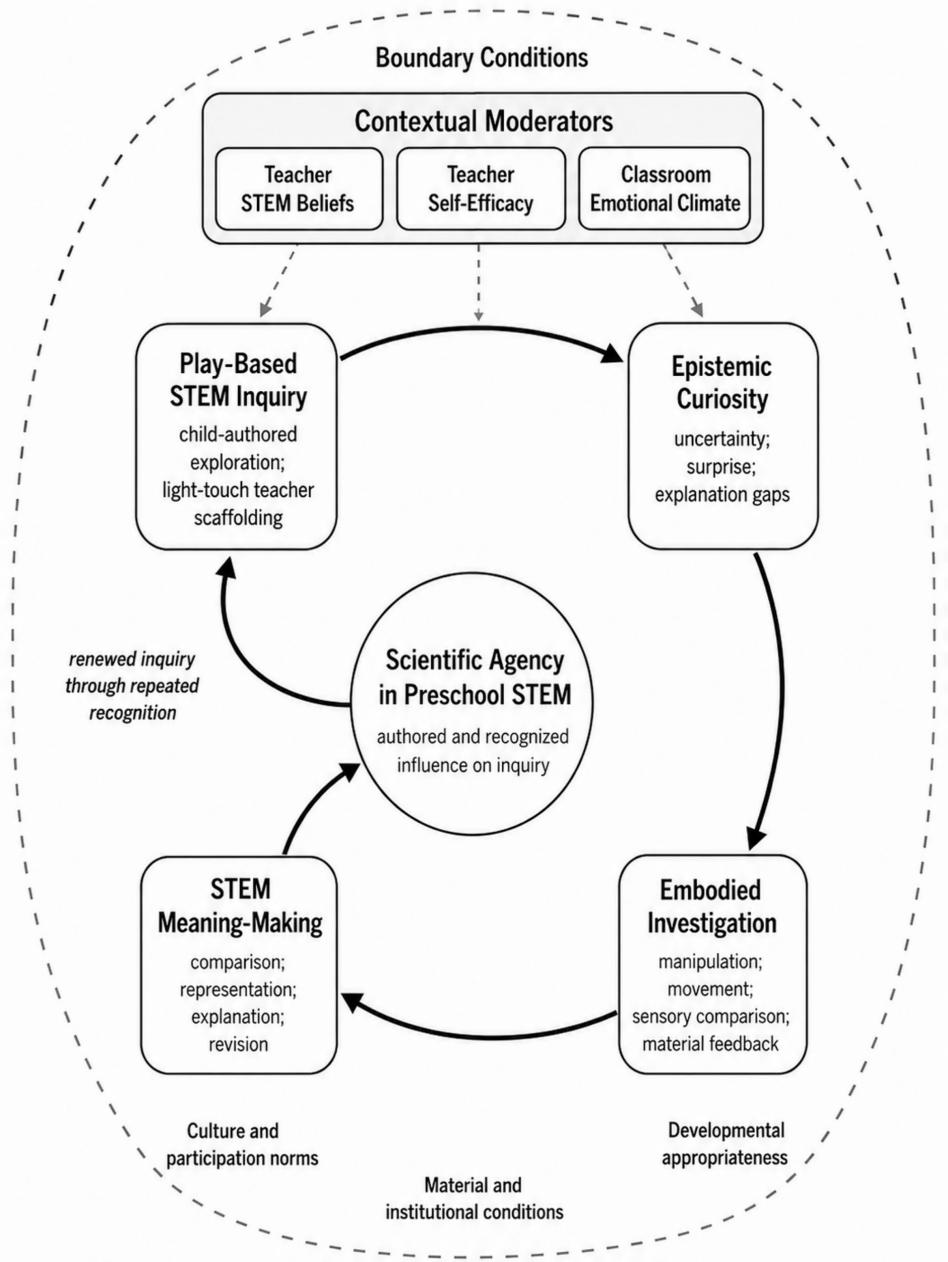
The play-based STEM agency model.

The model is theoretically derived rather than empirically validated as a complete system. It organizes established constructs into distinct functional roles. Play-based STEM inquiry is the pedagogical entry condition. Epistemic curiosity is the motivational mediator. Embodied investigation is the investigative mechanism. STEM meaning-making is the interpretive mechanism. Scientific agency is the proximal outcome and recursive driver of further inquiry. Teacher STEM beliefs, teacher self-efficacy, and classroom emotional climate moderate the strength and quality of these relations. Culture, developmental appropriateness, and material/institutional conditions function as boundary conditions. This hierarchy is important because it prevents the framework from appearing as an accumulation of concepts (Figure 1).

The model represents preschool STEM learning as a recursive process in which play-based inquiry creates accessible uncertainty, epistemic curiosity motivates investigation, embodied action generates material feedback, and STEM meaning-making connects experience with comparison, representation, explanation, and revision. Scientific agency emerges when children’s contributions become consequential for the direction of inquiry and are recognized by teachers and peers. Teacher STEM beliefs, teacher self-efficacy, and classroom emotional climate function as contextual moderators, while culture, developmental appropriateness, and material/institutional conditions operate as boundary conditions.

## Play-based STEM inquiry as the pedagogical entry condition

Play-based STEM inquiry is the entry condition of the model because it provides a developmentally appropriate context in which children can retain authorship while adults design the conditions for inquiry. It differs from both direct instruction and unframed free play. Direct instruction can introduce vocabulary or procedures efficiently, but it may reduce uncertainty and limit children’s influence on inquiry. Unframed free play preserves autonomy, but its epistemic direction may remain diffuse. Play-based STEM inquiry seeks a balance: children experience ownership over action and meaning, while adults arrange materials, time, discourse, and documentation so that comparison, evidence, and explanation become possible.

Guidance in this model does not mean controlling children’s play or leading them toward a single predetermined answer. It means designing epistemic affordances that make relations visible while preserving children’s authorship. A teacher might provide balls, cylinders, textured fabric, smooth boards, and measuring marks in a ramp area and ask, “What do you notice about how far these travel?” This question functions as guidance because it directs attention toward comparison without specifying which object to choose, which outcome to produce, or which explanation to accept. Children retain authorship by selecting materials, changing surfaces, proposing trials, repeating actions, and interpreting outcomes.

This distinction is crucial for preschool STEM. The teacher guides the ecology of inquiry, not every move of the child. Guidance may include selecting contrasting materials, arranging a problem space with multiple possible strategies, asking an open question, documenting children’s ideas, revoicing an informal hypothesis, or returning to an unresolved problem later. Such support keeps playful activity epistemically productive without converting it into a hidden worksheet or adult-controlled demonstration. This position is consistent with work showing both the value of guided play and the risks of overly directive instruction that can limit spontaneous exploration (Alfieri et al., 2011; Bonawitz et al., 2011; Fisher et al., 2013; Hirsh-Pasek et al., 2009; Weisberg et al., 2013; Zosh et al., 2017).

## Epistemic curiosity as the motivational mediator

Epistemic curiosity is the motivational mediator in the model. Curiosity can mean general attraction to novelty, but preschool STEM requires a more specific form of knowledge-seeking motivation. Novelty curiosity refers to children’s attraction to something new or perceptually interesting. Situational interest refers to temporary engagement generated by an activity, material, or social context. Epistemic curiosity refers to children’s motivation to reduce an explanation gap, resolve uncertainty, or understand why something happened (Engel, 2011; Jirout and Klahr, 2012; Loewenstein, 1994).

In this model, epistemic curiosity does not mean that a child simply likes a material. It means that the child treats uncertainty as something to investigate. A tower collapses although it looked stable. One ball rolls farther than another. A seed grows more near the window. A shadow changes when the lamp moves. These events can invite children to ask why, repeat a trial, compare conditions, or propose an explanation. Epistemic curiosity therefore connects play-based inquiry to scientific agency: it gives children a reason to initiate or continue inquiry, test an idea, compare outcomes, ask for another trial, or revise an explanation.

The mediating role of epistemic curiosity is important for the empirical logic of the model. Play-based inquiry is expected to support scientific agency partly because it creates manageable uncertainty that children want to pursue. Without curiosity, play may remain enjoyable but conceptually diffuse. Without teacher mediation and emotional safety, uncertainty may become frustrating or threatening. Epistemic curiosity therefore links the design of the activity to children’s inquiry behavior. In hypothesis-testing terms, play-based inquiry is expected to increase epistemic curiosity, epistemic curiosity is expected to increase inquiry behavior, and repeated inquiry behavior is expected to contribute to scientific agency.

## Embodied investigation as the investigative mechanism

Embodied investigation is the mechanism through which children test possibilities in material form. Preschool children often reason through perception and action before they can fully articulate their thinking. They push, pour, stack, rotate, align, sort, count, tilt, gesture, and repeat actions to explore relations among objects and outcomes. Embodied and grounded cognition perspectives suggest that conceptual activity is situated in perception, movement, and material contexts (Barsalou, 2008; Wilson, 2002). In preschool STEM, bodily and material action is not secondary to thinking; it is one way thinking becomes visible and revisable.

This claim also requires a sociocultural account of interpretation. Vygotsky’s theory emphasizes that children’s development is mediated through tools, language, interaction, and culturally organized activity (Vygotsky, 1978). In preschool STEM, a child’s material action becomes inquiry when it is connected to shared attention, language, comparison, and evidence. A child may first understand through action: moving a block, changing a ramp surface, rotating an object, or repeating a trial. The teacher may then make that action visible through revoicing, questioning, or naming. Peers may imitate, challenge, extend, or modify the action. The material world also contributes feedback: the tower falls, the marble slows, the water leaks, the bridge bends, or the plant fails to grow as expected.

Embodied action becomes interpretable when material feedback is taken up by the child, named or revoiced by the teacher, and made available to peers as something that can be compared, discussed, or tested again. Interpretation is therefore distributed across child, teacher, peers, and materials. The child interprets through action, attention, gesture, and repeated attempts. The teacher interprets by noticing, naming, questioning, and connecting action to language or representation. Peers interpret by imitating, extending, challenging, or modifying the action. This process transforms individual manipulation into shared evidence-making.

Embodied investigation is not automatically productive. Children can manipulate materials repetitively without comparing outcomes or constructing explanations. Teacher support matters, but it should be contingent and light. Teachers can invite prediction before acting, slow observation after a surprising result, ask children to compare two outcomes, revoice a child’s informal theory, or support documentation through drawing, photographs, tally marks, gesture, or shared records. The goal is not to interrupt play, but to help action become evidence.

## STEM meaning-making as the interpretive mechanism

STEM meaning-making is the interpretive mechanism of the model. It refers to the process through which children connect material action and observed outcomes to emerging explanatory, representational, and comparative relations. It includes comparing, classifying, predicting, measuring, representing, explaining, and revising. A child who says, “This one went farther because the board is smoother,” is coordinating action, evidence, comparison, and explanation. A child who draws the taller plant near the window, counts how many blocks a bridge can hold, or sorts objects by whether they float is moving from experience toward representation.

Three mechanisms are central. First, comparison helps children notice relations among actions, materials, and outcomes. They may compare two ramps, two towers, two bridge designs, two containers, or two plant locations. Second, representation allows children to stabilize and revisit experience through drawing, gesture, photographs, marks, numbers, charts, maps, or shared records. Third, explanation and revision help children connect observed outcomes to possible causes and modify their ideas when evidence changes. These processes are consistent with early science education accounts that emphasize observing, predicting, comparing, representing, explaining, and using evidence as central practices in science learning (Gelman et al., 2009; National Research Council, 2007, 2012; Worth, 2010).

This component is crucial because hands-on activity is not self-interpreting. The same materials can support entertainment, social play, motor practice, or conceptual learning depending on how children and teachers frame, discuss, document, and revisit the experience. Inquiry becomes STEM meaning-making when children are supported to connect what they did with what happened and why it might have happened. Preschool implementation must keep these practices flexible and developmentally appropriate rather than turning inquiry into a rigid procedure.

Teacher discourse is especially consequential. Open-ended questions, contrastive prompts, revoicing, elaborative feedback, and invitations to predict before acting can help children move from everyday noticing toward more systematic comparison and explanation (Studhalter et al., 2021; Worth, 2010). Representational supports such as drawings, photographs, gestures, charts, maps, and shared records can also consolidate meaning by making children’s ideas visible across time. Scientific language may be useful, but terminology should not replace sense-making. A teacher can add language to a child’s idea without taking authorship away: “You noticed that the tall block helps hold the bridge. In building, that part is supporting the bridge.”

## Scientific agency as proximal outcome and recursive driver

Scientific agency is the model’s central proximal outcome, but it also functions recursively. It emerges when children experience their own questions, actions, explanations, and revisions as consequential for inquiry. A child who says, “Let’s make it wider this time,” after a bridge fails is not simply participating. The child is using evidence from failure to redesign the next trial. Scientific agency is visible because the child’s idea changes what happens next.

In classroom terms, scientific agency can be inferred when children generate questions, initiate tests or modifications, interpret outcomes, use failure as information, revise explanations, or influence the group’s next step. It is also visible when a teacher or peer takes up a child’s idea as worthy of investigation. For example, a child’s observation that one object rolls farther may lead the group to compare surfaces. A child’s failed tower design may become the basis for testing wider foundations. A child’s question about why one plant grew more may lead to a new investigation of light, water, or location.

The recursive role of agency is equally important. When children repeatedly experience that their questions lead somewhere, that their trials generate evidence, and that their revisions are recognized, they are more likely to initiate future inquiry. Agency develops through repeated recognition. A child asks a question and the class investigates it. A child proposes a test and peers try it. A child revises an explanation and the teacher treats that revision as intelligent rather than wrong. Over time, these episodes can support a broader stance toward the world as investigable and toward oneself as a capable participant in inquiry.

The model therefore treats scientific agency as both an outcome of prior inquiry and a driver of renewed inquiry. This is why the model figure is recursive rather than linear. Play-based inquiry may lead to curiosity, embodied investigation, and meaning-making; however, once children’s contributions are recognized, agency feeds back into new questions, new tests, and new forms of participation.

## Contextual moderators and boundary conditions

The model identifies three contextual moderators: teacher STEM beliefs, teacher self-efficacy, and classroom emotional climate. These moderators do not replace the core mechanism chain. Rather, they shape whether play-based STEM inquiry becomes epistemically productive and whether children’s curiosity, embodied action, and meaning-making consolidate into scientific agency.

## Teacher STEM beliefs

Teacher STEM beliefs influence scientific agency through the way teachers treat uncertainty, talk, error, and children’s ideas. The mechanism can be summarized as follows: beliefs about STEM shape the treatment of uncertainty; the treatment of uncertainty shapes teacher discourse; teacher discourse shapes child authorship; and child authorship shapes scientific agency. Teachers who view STEM mainly as correct-answer knowledge may close inquiry early by giving answers, correcting quickly, or narrowing possible strategies. Teachers who view STEM as collaborative sense-making are more likely to preserve uncertainty, invite comparison, and treat failed attempts as opportunities for revision.

This distinction matters because preschool STEM depends on how teachers interpret children’s partial ideas. A child’s uncertain explanation can be treated as wrong, ignored, corrected, or used as the beginning of inquiry. Inquiry-oriented beliefs make it more likely that teachers ask children what they noticed, why they think something happened, what they want to try next, or how they know a new design worked better. Research on early childhood STEM highlights the importance of teacher beliefs, pedagogical content knowledge, and professional learning for shaping children’s learning opportunities (Campbell et al., 2018; Chen et al., 2025; MacDonald et al., 2020; Wan et al., 2021).

## Teacher self-efficacy

Teacher self-efficacy refers to teachers’ beliefs about their capacity to support STEM learning effectively. In this model, self-efficacy is not merely general confidence. It concerns whether teachers feel able to respond productively to unexpected child questions, tolerate open-ended inquiry, make pedagogical decisions under uncertainty, and use failed outcomes as opportunities for learning. Teachers who feel uncertain about science, engineering, or mathematics may avoid open-ended tasks, rely on heavily scripted routines, or close inquiry prematurely. Stronger self-efficacy may help teachers remain responsive when children ask unexpected questions or when outcomes do not follow the planned direction.

Content confidence alone is insufficient. A teacher may know scientific facts but still struggle to support preschool inquiry if uncertainty feels pedagogically risky. Conversely, a teacher with modest content knowledge may support meaningful inquiry by preserving children’s questions, inviting comparison, and documenting emerging explanations. The key issue is whether teachers feel capable of sustaining inquiry while guiding children toward evidence, representation, and explanation (Riggs and Enochs, 1990; Zee and Koomen, 2016).

## Classroom emotional climate

Classroom emotional climate is a central moderator because uncertainty is affectively risky. Children must feel safe enough to offer incomplete explanations, tolerate failure, and allow their ideas to become publicly discussable. The mechanism can be stated as follows: epistemic uncertainty creates affective risk; emotional safety and error normalization reduce that risk; reduced risk supports persistence and idea-sharing; and persistence and idea-sharing support scientific agency.

This construct should be understood as measurable rather than merely atmospheric. Indicators may include positive climate, teacher sensitivity, responsiveness to children’s ideas, normalization of mistakes, absence of ridicule, equitable turn distribution, and encouragement to try again after unsuccessful attempts. Observational systems such as the Classroom Assessment Scoring System and related interactional coding approaches provide possible starting points for measuring emotional support, teacher sensitivity, and instructional interactions (Hamre et al., 2013; Pianta et al., 2008).

Emotional climate moderates the model because uncertainty does not automatically produce curiosity. In an emotionally safe classroom, a collapsed tower can become a reason to ask what changed. In a rigid or evaluative classroom, the same collapse may become a reason to stop, hide the failure, or wait for the teacher’s answer. The model therefore predicts that uncertainty supports inquiry most strongly when children experience the classroom as a place where partial ideas, mistakes, and revisions are legitimate.

## Boundary conditions

Three boundary conditions shape how the model can be enacted: culture and participation norms, developmental appropriateness, and material/institutional conditions.

Culture shapes norms about play, adult authority, questioning, error, peer talk, and appropriate participation. A form of teacher mediation that supports agency in one cultural setting may require adaptation in another. Some communities may emphasize collective participation, observation before speaking, respect for adult authority, or indirect forms of questioning. These differences do not make scientific agency irrelevant, but they require researchers and educators to examine how agency is recognized in culturally responsive ways. Funds-of-knowledge perspectives are especially useful here because they remind educators that children’s everyday experiences, family practices, and community knowledge can become resources for classroom inquiry (Moll et al., 1992).

Developmental appropriateness is also a boundary condition. Preschool STEM should not be defined by adding more disciplinary labels or by moving later school content downward. It should be defined by selecting phenomena, tools, representations, and questions that children can perceive, manipulate, discuss, represent, and revisit. A STEM topic is developmentally appropriate when children can act on relevant relations and participate meaningfully in sense-making. If a task depends mainly on abstract terminology or adult-controlled procedure, scientific agency is likely to be limited.

Material and institutional conditions shape how fully the model can be enacted. Time, space, staffing ratios, access to materials, documentation routines, curriculum expectations, and assessment pressures all influence whether children have repeated opportunities for inquiry and revision. A teacher may endorse inquiry-oriented STEM but lack the time, support, or resources to sustain it. The model should therefore not be interpreted as a purely individual theory of child agency. Scientific agency is also produced or constrained by the cultural, institutional, and material arrangements of early childhood settings.

In summary, play-based STEM inquiry supports scientific agency through epistemic curiosity, embodied investigation, and STEM meaning-making. Teacher beliefs, teacher self-efficacy, and classroom emotional climate moderate this process, while culture, developmental appropriateness, and material/institutional conditions define the contexts in which the process can unfold.

## Distinguishing the model from existing approaches

The Play-Based STEM Agency Model builds on established approaches rather than replacing them. Its contribution is not the claim that play, curiosity, embodiment, inquiry, teacher mediation, or emotional climate are new constructs. Its contribution lies in organizing these constructs into a mechanism-focused account of how preschool STEM experiences become child-authored inquiry. The model specifies what each approach contributes, what it leaves underexplained for preschool STEM agency, and what the present framework adds.

Constructivist and sociocultural accounts provide the broadest theoretical foundation. Constructivist perspectives emphasize that children actively construct knowledge through manipulation, exploration, cognitive conflict, and reorganization of understanding (Piaget, 1952). Sociocultural perspectives emphasize that learning is mediated through tools, language, interaction, and participation in culturally organized activity (Vygotsky, 1978). These traditions explain why action and social mediation matter. Their limitation for the present problem is that they do not, by themselves, specify when preschool STEM activity becomes agency-supporting inquiry. The model adds a more focused account of how curiosity, embodied investigation, meaning-making, teacher mediation, and recognition work together around scientific agency.

Play-based learning and guided play research show that adult support and child autonomy can coexist. This literature is important because it challenges the false opposition between child-initiated play and adult-supported learning (Hirsh-Pasek et al., 2009; Pyle et al., 2017; Weisberg et al., 2013; Zosh et al., 2017). Recent meta-analytic evidence also suggests that guidance during play can support children’s learning, although effects vary by domain, implementation, and the form of adult support provided (Skene et al., 2022). Its limitation is that guidance is sometimes described broadly, without specifying when guided play becomes epistemically productive STEM inquiry. The model’s addition is to explain this transformation through accessible uncertainty, epistemic curiosity, embodied investigation, STEM meaning-making, and recognized child authorship.

Inquiry-based science education identifies important practices such as observing, predicting, testing, representing, explaining, and using evidence (National Research Council, 2007, 2012). These practices are essential for STEM learning. However, inquiry can become a procedural checklist if implemented rigidly: ask a question, make a prediction, conduct an experiment, record results, and draw a conclusion. In preschool, such rigidity can reduce developmental appropriateness and child authorship. The present model shifts the focus from whether inquiry steps were completed to whether children participated agentically in generating, sustaining, and interpreting inquiry.

Integrated STEM pedagogy emphasizes interdisciplinary connections, design challenges, teacher pedagogical content knowledge, and professional learning (Campbell et al., 2018; MacDonald et al., 2020; Wan et al., 2021). This literature helps explain how science, technology, engineering, and mathematics can be connected in early childhood settings. Its limitation is that integration across domains does not automatically specify children’s epistemic role within inquiry. The model adds a child-level psychological mechanism by asking how teacher-designed experiences become meaningful for preschool children through curiosity, embodied investigation, representation, explanation, and agency. Integration is valuable only when it remains coherent, developmentally appropriate, and open to child authorship.

Science identity frameworks illuminate recognition, competence, performance, and belonging as important dimensions of participation in science (Carlone and Johnson, 2007). This literature is valuable because it shows that participation in science is not only cognitive but also social and identity-related. Its limitation for preschool STEM is developmental scale. Preschool children may meaningfully contribute to inquiry episodes before developing stable self-descriptions as science learners or longer-term narratives of belonging in science. Scientific agency is therefore positioned as a proximal, episode-level form of epistemic authorship that may contribute to later science identity.

The model also clarifies the role of embodiment and emotional climate. Many early STEM accounts endorse hands-on activity, but hands-on activity alone is not the mechanism. Embodied investigation matters because action on materials helps children discover variables, externalize reasoning, receive feedback, and coordinate experience with representation. Similarly, emotional climate matters because uncertainty, failure, and revision are emotionally demanding. A child who fears being wrong is less likely to offer a tentative explanation or redesign a failed structure. Emotional climate is therefore not an optional background condition; it is part of the ecology that makes agency possible.

These distinctions answer the likely question of whether the model is simply another version of child-centered, play-based inquiry learning. It is not. The model integrates familiar components around a more specific outcome and a more explicit mechanism chain. It asks how preschool classrooms create conditions in which children’s actions, questions, explanations, and revisions become consequential for inquiry. Its novelty lies in explaining the development of scientific agency in preschool STEM through the combined roles of play, uncertainty, embodied action, interpretation, mediation, recognition, and context.

## Testable hypotheses

A conceptual model is useful for research when it generates clear empirical expectations. In this article, empirical expectations refer to observable and testable predictions about relations among pedagogy, curiosity, embodied action, teacher discourse, classroom climate, and child agency. The Play-Based STEM Agency Model therefore advances six hypotheses (Table 1). These hypotheses are broad enough to guide experimental, observational, longitudinal, and mixed-method research, yet specific enough to support operationalization in preschool settings.

Hypothesis 1: Play-based STEM inquiry with light-touch scaffolding will elicit higher epistemic curiosity than direct instruction or unframed free play on comparable content.

This hypothesis follows from the claim that play-based STEM inquiry preserves meaningful uncertainty while providing sufficient structure to make exploration tractable. Direct instruction may reduce the information gap that triggers curiosity by telling children what to notice and conclude. Unframed free play may allow activity but fail to orient attention toward evidence or explanation. Play-based STEM inquiry should therefore increase question-asking, anomaly-focused attention, requests for additional trials, and time spent exploring unresolved contrasts.

Hypothesis 2: Epistemic curiosity will mediate the relation between play-based STEM inquiry and children’s inquiry behavior.

This hypothesis clarifies the meaning of mediation. Play-based STEM inquiry is expected to increase epistemic curiosity by creating manageable uncertainty. Epistemic curiosity is then expected to increase inquiry behavior by motivating children to ask explanation-oriented questions, test alternatives, compare outcomes, revise ideas, and seek further evidence. The mediational claim is that play-based inquiry supports inquiry behavior partly because it generates curiosity about uncertainty, not merely because it increases activity level. This distinction separates inquiry behavior from general participation.

Hypothesis 3: Agency-promoting teacher discourse will positively predict children’s scientific agency.

Agency-promoting discourse includes open-ended questions, revoicing of child ideas, contrastive prompts, invitations to predict before acting, elaborative feedback, and teacher uptake of child-generated hypotheses. Such discourse positions children as contributors to meaning-making rather than as responders to adult prompts alone. It should predict child-initiated testing, explanation attempts, revisions, and the likelihood that a child’s idea changes the next step in the activity.

Hypothesis 4: Embodied investigation paired with teacher scaffolding will predict stronger STEM meaning-making and scientific agency than verbally equivalent explanation without child manipulation.

This hypothesis connects embodiment directly to the model. If embodied investigation is central, children who manipulate materials, receive feedback, and discuss their actions should show stronger explanation, representation, transfer, and agency indicators than children who only hear an explanation or watch a demonstration. The hypothesis does not claim that action alone is sufficient. The predicted advantage depends on scaffolding that helps children coordinate action with comparison and interpretation.

Hypothesis 5: Teacher STEM self-efficacy and inquiry-oriented beliefs will moderate the quality of children’s scientific agency.

Classrooms led by teachers with stronger STEM self-efficacy and more inquiry-oriented beliefs should provide more favorable conditions for scientific agency, even when materials are similar. Such teachers are expected to preserve uncertainty longer, respond more productively to unexpected child questions, and frame failed attempts as opportunities for revision. Multilevel designs can test this hypothesis by linking teacher surveys and classroom observations to child agency indicators.

Hypothesis 6: Classroom emotional climate will moderate the relation between epistemic uncertainty and scientific agency.

The model predicts that uncertainty supports inquiry most strongly when classroom climate is emotionally safe. In classrooms characterized by positive climate, teacher sensitivity, error normalization, and equitable participation, children should be more willing to offer tentative ideas and persist after failure. In more evaluative or rigid climates, the same uncertainty may reduce participation or increase dependence on the teacher.

**Table 1:** Summary of hypotheses and possible empirical indicators.

Hypothesis	Core prediction	Possible empirical indicators
H1	Play-based STEM inquiry with light-touch scaffolding elicits higher epistemic curiosity than direct instruction or unframed free play.	Child questions; anomaly-focused talk; exploration time; requests for further trials; attention to unresolved contrasts.
H2	Epistemic curiosity mediates the relation between play-based STEM inquiry and inquiry behavior.	Explanation-oriented questions; variable testing; repeated trials; comparison; revision; evidence-seeking.
H3	Agency-promoting teacher discourse predicts scientific agency.	Open prompts; revoicing; contrastive questions; elaborative feedback; teacher uptake of child ideas; child-initiated tests.
H4	Embodied investigation plus scaffolding predicts stronger STEM meaning-making and scientific agency.	Material manipulation; gesture; drawings; explanations; redesigns; transfer tasks; evidence-based revisions.
H5	Teacher STEM self-efficacy and inquiry-oriented beliefs moderate children’s scientific agency.	Teacher surveys; belief–practice alignment; discourse coding; classroom observations; multilevel child outcomes.
H6	Classroom emotional climate moderates the relation between uncertainty and agency.	Positive climate; teacher sensitivity; error normalization; equitable turns; persistence after failure; willingness to share tentative ideas.

## Implications for future research

The Play-Based STEM Agency Model suggests a research agenda organized around three priorities: measurement, design, and scope. The central empirical task is not only to determine whether preschool STEM activities improve outcomes, but to examine how classroom conditions shape children’s epistemic roles, inquiry trajectories, and opportunities for recognized participation.

## Measurement priorities

A first priority is the development of measures that capture scientific agency as a situated, multimodal, and interactional construct. Existing studies often include useful indicators such as question-asking, engagement, curiosity, science process skills, or content vocabulary. These indicators are valuable, but they do not by themselves show whether children influence the direction or interpretation of inquiry. Future observational systems should therefore distinguish general participation from agency-relevant participation.

Scientific agency should be measured through multiple forms of evidence. Relevant indicators include child-generated questions, child-initiated tests, material action, gesture, peer uptake, teacher uptake, evidence-based revision, and persistence after failure. These indicators are especially important in preschool because reasoning is often expressed through action before it is expressed through formal language. A child who changes the base of a tower after collapse, repeats a ramp trial with a different surface, points to a relevant contrast, or gestures the movement of a shadow may be showing inquiry-relevant reasoning even without extended verbal explanation.

Measurement should also capture whether children’s contributions become consequential. A child’s question may be ignored, briefly acknowledged, or used to organize the next investigation. A teacher may revoice a child’s idea in a way that preserves the child’s authorship, or may appropriate the idea and redirect the task toward an adult-controlled answer. Peer uptake matters as well. When one child’s observation is imitated, challenged, extended, or tested by other children, the observation becomes part of a shared inquiry trajectory. Coding systems should therefore examine not only what children say or do, but also how their contributions are taken up by teachers and peers.

Video-based microgenetic methods are particularly well suited to this work. Fine-grained video analysis can show how inquiry develops across seconds and minutes: how a puzzling event becomes a question, how a teacher prompt changes the direction of activity, how a gesture becomes a shared reference point, and how a failed attempt becomes evidence for redesign. Such analysis can also reveal moments when agency is curtailed, for example when a teacher closes uncertainty too quickly, corrects before children compare alternatives, or values only the most verbal children’s ideas.

Curiosity also requires sharper measurement. Future research should distinguish novelty curiosity, situational interest, and epistemic curiosity. Novelty curiosity may appear as attraction to a new object or material. Situational interest may appear as temporary engagement in an enjoyable activity. Epistemic curiosity should be coded through explanation-oriented behavior: why/how questions, attention to anomalies, requests for additional trials, persistence with unresolved contrasts, and attempts to compare possible causes. This distinction is necessary because the model predicts that epistemic curiosity, not general activity or enjoyment alone, mediates the relation between play-based inquiry and scientific agency.

Teacher-level and classroom-level constructs should be measured alongside child behavior. Self-report measures of teacher beliefs and self-efficacy remain useful, but they should be paired with video observation, discourse analysis, stimulated recall, and classroom climate measures. Belief–practice alignment is especially important. A teacher may endorse inquiry in principle while still closing inquiry prematurely in practice. Classroom emotional climate should also be measured through observable indicators such as teacher sensitivity, positive climate, error normalization, equitable turn distribution, and responsiveness to children’s partial ideas (Hamre et al., 2013; Pianta et al., 2008).

## Design priorities

A second priority concerns research design. Experimental and quasi-experimental studies can test the model by comparing play-based STEM inquiry, direct instruction, and unframed free play under controlled conditions. These comparisons should use the same materials, the same focal concept, the same broad learning goal, and comparable time-on-task. Such controls are important because they isolate pedagogical framing rather than confounding pedagogy with content, materials, or activity quality.

For example, a study on ramps and motion could compare three conditions. In a direct instruction condition, the teacher explains the target relation and demonstrates the procedure. In an unframed free play condition, children freely use the same ramps and objects without structured prompts. In a play-based inquiry condition, children use the same materials while the teacher preserves choice, invites comparison, and supports representation without predetermining the answer. Outcomes should include epistemic curiosity, embodied investigation, STEM meaning-making, and scientific agency, rather than vocabulary recall alone.

Designs should also test the proposed mediational and moderating relations. Mediation analyses can examine whether play-based inquiry increases agency partly through epistemic curiosity and inquiry behavior. Moderation analyses can examine whether teacher beliefs, teacher self-efficacy, and classroom emotional climate strengthen or weaken these relations. Multilevel models are especially appropriate because child behavior is nested within classrooms, teachers, programs, and institutional settings.

Longitudinal research is also needed. Scientific agency is unlikely to develop through a single activity. Children need repeated opportunities to pose questions, act on materials, compare outcomes, revise ideas, and experience recognition. Longitudinal designs could examine whether agency indicators in preschool predict later science motivation, persistence in problem solving, openness to uncertainty, or participation in kindergarten and early primary inquiry settings.

Design-based research with teachers would be particularly valuable. Such work can refine prompts, materials, documentation practices, and professional learning routines under realistic classroom constraints. Rather than testing only whether an intervention works under ideal conditions, design-based studies can examine how teachers adapt inquiry supports when time, space, staffing ratios, assessment expectations, and curriculum requirements vary.

## Scope priorities

A third priority concerns scope. The model should be examined across cultural, linguistic, institutional, and socioeconomic contexts rather than assumed to operate identically everywhere. Norms about play, adult authority, questioning, peer talk, error, and public explanation vary across communities. This is especially important because STEM agendas and educational priorities are shaped differently across Global North and Global South contexts, including differences in resources, policy histories, equity goals, and institutional constraints (Mudaly and Chirikure, 2023). Future studies should therefore ask how scientific agency is recognized in different cultural settings, including Global South, non-Western, multilingual, and under-resourced early childhood contexts, and how culturally responsive forms of recognition and scaffolding can support children’s participation.

Equity requires particular attention. Scientific agency may be unevenly distributed if teachers respond more elaboratively to some children than others, if verbal fluency is mistaken for reasoning, or if some participation styles are more readily recognized as “scientific.” Research should examine how language background, disability status, gendered expectations, race, ethnicity, and socioeconomic context shape access to epistemic authority. Measures must be sensitive to children who reason through gesture, material manipulation, observation, imitation, delayed participation, or peer collaboration rather than extended verbal explanation.

Disability and neurodiversity should be included more explicitly in future work. Children with speech, language, sensory, motor, attentional, or social communication differences may show agency through alternative modalities. A child may participate by arranging materials, pointing, repeating a test, visually tracking a contrast, selecting a tool, or collaborating nonverbally with a peer. Measures that privilege verbal explanation alone risk underestimating such children’s agency.

Research should also extend beyond classroom settings. Home and community activities such as cooking, gardening, repairing objects, sorting materials, observing weather, caring for animals, building with household items, and discussing everyday technologies can provide meaningful contexts for early STEM inquiry. Funds-of-knowledge perspectives suggest that children’s family and community experiences can become resources for classroom sense-making when teachers recognize them as epistemically valuable (Moll et al., 1992).

Finally, future studies should address material and institutional constraints directly. Time, space, staffing, resource availability, documentation requirements, curriculum expectations, and assessment pressures shape which forms of inquiry are feasible. A model of preschool STEM that ignores these constraints risks overstating what teachers and children can realistically sustain. Research should therefore examine not only child-level indicators, but also the structural conditions that make scientific agency more or less possible.

## Discussion

This article has argued that preschool STEM should be understood not merely as early exposure to disciplinary content or as the provision of hands-on activities, but as a context for developing scientific agency. The proposed account places children’s inquiry-relevant contributions at the center of early STEM learning. In doing so, it shifts attention from activity labels to the processes through which children notice phenomena, act on materials, compare outcomes, revise explanations, and have their contributions recognized within classroom interaction.

The Play-Based STEM Agency Model contributes to early STEM scholarship by organizing familiar constructs into a more explicit mechanism chain. Play-based inquiry provides a developmentally appropriate entry condition. Epistemic curiosity explains why children pursue uncertainty. Embodied investigation explains how children test possibilities through material action. STEM meaning-making explains how action becomes connected to comparison, representation, explanation, and revision. Scientific agency names the point at which children’s contributions become consequential for the direction or interpretation of inquiry. Teacher beliefs, teacher self-efficacy, emotional climate, culture, developmental appropriateness, and material conditions shape whether this process can unfold.

The model should therefore not be read as a romantic defense of play or as a claim that child autonomy alone produces STEM learning. Its central claim is more conditional: play becomes STEM inquiry when uncertainty is preserved, action is made interpretable, and children’s contributions are recognized without abandoning conceptual guidance. This qualification is important because unguided discovery and loosely framed exploration do not reliably support learning. Without adequate guidance, children may manipulate materials extensively while missing relevant contrasts, overlooking evidence, or failing to connect action with explanation (Alfieri et al., 2011).

This creates a productive tension for preschool STEM. Too much adult control can reduce child authorship, close uncertainty, and convert inquiry into task completion. Too little structure can leave children with enjoyable but conceptually diffuse activity. The model responds to this tension by emphasizing light-touch scaffolding: teachers design affordances, preserve uncertainty, support comparison, and introduce language without predetermining every action or answer. Guidance is not opposed to agency; it becomes agency-supportive when it expands what children can notice, test, represent, and discuss.

A second tension concerns content mastery. Play-based STEM can risk shallow conceptual development if educators treat enjoyment, engagement, or activity completion as sufficient evidence of learning. Children may build, pour, sort, or draw without developing more stable understandings of relation, cause, evidence, quantity, structure, or design constraint. The proposed model does not reject conceptual goals. Instead, it argues that conceptual learning in preschool should be pursued through developmentally appropriate forms of inquiry: comparison, representation, explanation, revision, and shared interpretation. Vocabulary and content knowledge matter, but they should be connected to children’s actions and observations rather than imposed as isolated terms.

A third tension concerns teacher workload. Open-ended inquiry can be demanding. Teachers must manage materials, safety, peer interaction, documentation, assessment, and unexpected questions while preserving children’s initiative. They must decide when to intervene, when to wait, when to ask a question, and when to introduce a term or representation. These demands are substantial, especially in classrooms with limited staffing, large group sizes, rigid schedules, or high assessment pressure. The model therefore should not be interpreted as placing responsibility only on individual teachers. Supporting scientific agency requires professional learning, planning time, accessible materials, documentation tools, and institutional recognition that inquiry is legitimate early childhood pedagogy.

A fourth tension concerns institutional constraints. Curriculum standards, school readiness expectations, limited materials, time pressures, and accountability systems may push teachers toward visible products and easily documented outcomes. In such contexts, STEM can become a craft, worksheet, demonstration, or scripted activity rather than inquiry. The model challenges these tendencies by treating uncertainty, revision, and child contribution as central features of meaningful STEM learning. However, this challenge is practical as well as theoretical. Programs need structures that allow children to revisit questions, repeat trials, document thinking, and learn from failed attempts.

A fifth tension concerns equity. Scientific agency is socially recognized, and recognition is never neutral. Verbally confident children may receive more uptake. Children from dominant language backgrounds may be more easily interpreted as knowledgeable. Quiet children, multilingual learners, children with disabilities, and children whose participation is primarily gestural, observational, or material may have their contributions overlooked. Gendered, racialized, cultural, and socioeconomic expectations may also shape which children are positioned as capable investigators. For this reason, scientific agency must be assessed through multimodal and culturally responsive indicators, not only through verbal explanation or teacher-selected participation.

These tensions sharpen rather than weaken the model’s contribution. They show that preschool STEM agency is not produced simply by adding STEM materials, encouraging free exploration, or using inquiry language. It depends on the interaction among pedagogy, motivation, embodiment, discourse, emotional safety, and recognition. The same activity can support very different forms of learning depending on how teachers frame uncertainty, how children’s ideas are taken up, and how the classroom treats error and revision.

The model also has implications for teacher education. Preparing teachers for preschool STEM should involve more than supplying activity ideas or increasing content knowledge. Teachers need opportunities to practice contingent guidance: asking open questions, revoicing children’s ideas, supporting comparison, documenting inquiry, and deciding when to introduce concepts without replacing child reasoning. Professional learning should also address beliefs about uncertainty, failure, and children’s competence. Teachers who view STEM as collaborative sense-making may be more likely to preserve inquiry long enough for children to compare, revise, and explain.

Assessment practices also need revision. If scientific agency is a meaningful developmental outcome, assessment should include process-sensitive indicators such as child-generated questions, child-initiated tests, evidence-based comparisons, peer uptake, teacher uptake, and revisions after discrepant outcomes. Content vocabulary and performance tasks may remain useful, but they cannot capture the full quality of preschool STEM participation. A child who redesigns a structure after failure or proposes a new test after an unexpected result may be demonstrating a form of learning that conventional assessments miss.

Overall, the framework positions preschool STEM as a present-tense educational experience, not only preparation for later schooling. Its value lies in helping children experience the world as investigable and themselves as capable contributors to inquiry. That value is realized only under particular conditions: when teachers preserve manageable uncertainty, when materials afford meaningful action, when representations support interpretation, when classrooms normalize revision, and when children’s contributions are recognized as consequential.

## Limitations

Several limitations should be stated clearly. First, the Play-Based STEM Agency Model has not yet been empirically tested as a complete system. Although its components are grounded in established scholarship on early childhood pedagogy, curiosity, embodiment, inquiry, teacher mediation, and agency, the proposed relations among these components remain theoretical. Future work must test whether the full mechanism chain operates as proposed and under what classroom conditions it is strongest.

Second, scientific agency may appear differently across STEM domains. Agency in early science may involve questioning, observing, testing, and explaining natural phenomena. Agency in engineering may involve designing, testing, failing, and redesigning. Agency in mathematics may involve patterning, quantifying, comparing, measuring, or representing relations. Agency in technology may involve tool use, sequencing, debugging, or evaluating designed systems. The present model treats STEM broadly, but future studies should examine whether domain-specific forms of agency require different indicators.

Third, the preschool focus requires developmental caution. This article uses preschool primarily to refer to children between approximately 3 and 6 years of age, while recognizing that early childhood is often defined more broadly. Preschool, kindergarten, and early primary contexts should not be treated as developmentally interchangeable. Children’s language, motor control, symbolic representation, peer collaboration, and metacognitive capacities change rapidly across these years. Future studies should specify age ranges carefully and avoid generalizing from older children to preschool learners without evidence.

Fourth, cross-cultural validity cannot be assumed. The model proposes general mechanisms, but the recognition of agency is culturally and institutionally mediated. Norms about adult authority, questioning, error, peer collaboration, and public explanation vary across communities. This is especially important because STEM agendas and educational priorities are shaped differently across Global North and Global South contexts, including differences in resources, policy histories, equity goals, and institutional constraints (Mudaly and Chirikure, 2023). A participation pattern interpreted as agency in one setting may appear differently in another. Cross-cultural and Global South research is therefore necessary before the model can claim broad applicability.

Fifth, teacher workload and institutional feasibility require further investigation. The model assumes that teachers can preserve uncertainty, document inquiry, respond contingently, and support repeated investigation. In practice, these actions require time, space, materials, planning, professional support, and manageable group sizes. Without institutional support, the model may be difficult to enact consistently. Future research should therefore examine implementation feasibility, not only theoretical desirability.

Sixth, measurement tools may privilege verbal children. Scientific agency can be expressed through speech, but also through gesture, material action, gaze, repetition, imitation, redesign, and peer collaboration. Instruments that rely heavily on verbal explanation may underestimate the agency of multilingual learners, children with speech or language differences, children with disabilities, younger preschoolers, and children whose participation is quieter or more observational. Multimodal measurement is therefore not only methodologically useful but also an equity concern.

Seventh, the long-term relation between scientific agency and science identity remains empirically uncertain. The model treats scientific agency as a proximal, episode-level form of epistemic authorship that may contribute to later science identity. However, whether repeated experiences of preschool STEM agency predict later science identity, STEM interest, persistence, or disciplinary participation remains an open empirical question. Longitudinal research is needed to examine how early agency episodes accumulate, stabilize, or transform across later educational contexts.

These limitations define the next stage of research. The model should be treated as a theoretically grounded framework for empirical testing, refinement, and contextual adaptation rather than as a completed account of preschool STEM learning. Its usefulness will depend on whether future studies can show when, for whom, and under what conditions play-based STEM inquiry supports scientific agency.

## Data Availability

The original contributions presented in the study are included in the article/supplementary material, further inquiries can be directed to the corresponding author/s.
